# Homozygosity for the Mediterranean α-thalassemic deletion (hemoglobin Barts hydrops fetalis)

**DOI:** 10.4103/0256-4947.60523

**Published:** 2010

**Authors:** Nasir A. S. Al-Allawi, Maida Y. Shamdeen, Najeeb S. Rasheed

**Affiliations:** a From the Department of Pathology, College of Medicine, University of Dohuk, Dohuk, Iraq; b From the Department of Gynecology and Obstetrics, College of Medicine, University of Dohuk, Dohuk, Iraq; c From the Department of Hematology, Azadi Teaching Hospital, Dohuk, Iraq

## Abstract

Hemoglobin Barts hydrops fetalis syndrome is the most severe and generally fatal clinical phenotype of α-thalassemia. We diagnosed a fetus at 23-weeks gestation with having hydrops fetalis, by ultrasound. At 32 weeks, intrauterine death was detected. Molecular studies revealed that the fetus had the hemoglobin Barts hydrops fetalis syndrome due to homozygosity for the Mediterranean α-thalassemia deletion. This clinical phenotype is generally rare in the Eastern Mediterranean, and this is the first report of this syndrome from Iraq. Techniques for molecular characterization became available only very recently in this country, in a diagnostic setting. Thus, the detection of further cases might be expected in future.

Hemoglobin Barts hydrops fetalis syndrome, the most severe and generally fatal clinical phenotype of a-thalassemia, is due to the deletion of all four functional α-globin genes of the hemoglobin, resulting in no α-globin chain production. This clinical phenotype is generally rare in the Eastern Mediterranean. This is the first case report of molecularly confirmed hemoglobin Barts hydrops fetalis from Iraq.

## CASE

A fetus was diagnosed with hydrops fetalis at 23-weeks gestation, by ultrasound, and intrauterine death was detected at 32 weeks; labor was induced and the fetus was delivered stillborn. The delivered stillborn had generalized edema, pallor, abdominal swelling, and hepatosplenomegaly. The placenta was large and friable. A full blood count performed on the cord blood using a Beckman-Coulter Hematology Analyzer (Fullerton, CA, USA) revealed a hemoglobin of 8.7 g/dL, a mean corpuscular volume of 94 fL, a mean corpuscular hemoglobin of 25 pg/cell, and a red blood cell (RBC) count of 3.46×10^12^/L. The Leishman-stained blood film revealed RBC hypochromasia, marked anisocytosis, numerous tear drops and target cells, and large numbers of nucleated red cells (150/100 white blood cells).

The mother was 23-year-old and had only one pregnancy, two years previously. This also ended with the delivery of a stillborn hydropic child after 30-weeks gestation. A similar incident could not be recounted in either the maternal or paternal family histories. During her current pregnancy, the blood pressure was consistently normal, and serial prenatal examinations did not reveal any proteinuria. EDTA-blood samples taken from the mother and the 22-year-old father, who was her first cousin, were used to obtain blood counts, for hemoglobin electrophoresis on cellulose acetate (pH 8.6), and to measure hemoglobin A_2_ by elution from cellulose acetate strips, and hemoglobin F by alkaline denaturation, using standard laboratory procedures.^1^ Both parents also had their serum iron, total iron binding capacity, and transferrin saturation determined (BioMérieux-Marcy l'Etoile, France) (Table 1).

**Table 1:** Some hematological parameters in parents of the hydropic child.

Parameter	Mother	Father
Hemoglobin (g/dL)	11.6	14.8
Hematocrit (L/L)	36.7	46.7
Mean corpuscular volume (fl)	63.3	67.9
Mean corpuscular hemoglobin (pg/cell)	20.0	21.5
Red cell distribution width (%)	15.9	15.7
Red cell count (×10^12^/L)	5.79	6.88
Hemoglobin A2 (%)	1.5	1.9
Hemoglobin F (%)	2.0	1.4
Transferrin saturation (%)	20	26
Blood group	AB Rh(D) positive	AB Rh(D) positive

**Figure 1:**
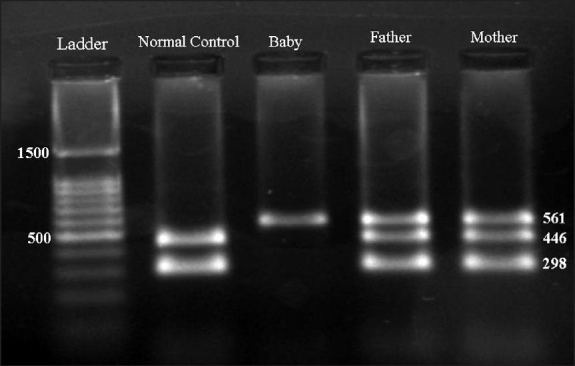
Gap-PCR for α-thalassemia deletions. The amplification products are run on 2% agarose gel. The normal (αα/αα) control shows two bands at 446 and 298 bp, while both parents show three bands (561, 446, and 298 bp) and are thus heterozygous for the Mediterranean deletion (αα/--^MED^). The infant shows only a single band at 561 bp and thus it is homozygous for the latter deletion (--^MED^/--^MED^).

The blood samples from the couple and their hydropic stillborn were then used for DNA extraction using a phenol/chloroform method, and the DNA was amplified in a gap polymerase chain reaction as previously described,^2^ to detect the Mediterranean (--^MED^) α-thalassemic deletion. The amplification products were then run on 2% agarose gel (Figure 1). Although both parents were heterozygous for (--^MED^) deletion (α-thalassemia minor phenotype), the stillborn child was homozygous for the deletion (--^MED^/--^MED^). This finding confirmed that the stillborn had hemoglobin Barts hydrops fetalis.

## DISCUSSION

The hemoglobin molecule is a tetramer of two pairs of globin chains, each containing a heme group. During fetal life the main hemoglobin is hemoglobin F (α2γ2), while in normal adults the main hemoglobin is hemoglobin A (α2β2). The production of α-globin chains of hemoglobin is directed by two functional alpha genes (α1 and α2) located in the α-globin gene cluster at the short arm of chromosome 16.^3^

α-thalassemia is an inherited disorder of hemoglobin synthesis, characterized by reduced or absent a-globin chain synthesis.^4^ It is caused by deletional or nondeletional mutations involving one (α^+^ defects) or both (α° defects) alpha genes *in cis* at the α gene cluster^5^. Different combinations of these two defects result in four clinical phenotypes of α-thalassemia. The silent carrier state is caused by heterozygosity to the α^+^ defect. α-thalassemia minor is caused by either homozygosity to the α^+^ defects or heterozygosity to the α° defects. Hemoglobin H disease is caused by compound heterozygosity to the α° and α^+^ defects, while homozygosity to the α° defect leads to the phenotype of hemoglobin Barts hydrops fetalis^3^. In the latter phenotype there is no production of a-chains, and therefore, the fetal hemoglobin (α2γ2) is not formed, but instead hemoglobin Barts is formed from four fetal γ chains (γ4). Hemoglobin Barts has a very high oxygen affinity, and will not be able to liberate oxygen at the physiological tissue tensions, and is thus ineffective as an oxygen carrier. Consequently, the fetus with this hemoglobin would have severe intrauterine hypoxia, leading to the intrauterine death of the hydropic fetus, late in pregnancy or at term.^4^

Several α° defects removing both alpha genes have been described in different populations, and they are named either in relevance to their geographical location or size. They include Southeast Asian (--^SEA^), Mediterranean (--^MED^), Philippino (--^FIL^), Thai (--^THAI^), and the 20.5 kb (-(α)^20.5^) variants.^4^,^6^ Hemoglobin Barts hydrops fetalis is most common in Southeast Asian (SEA) countries, where it is mainly due to homozygosity to an SEA deletion (--^SEA^/--^SEA^).^6^ It has also been reported, although uncommonly, in Mediterranean countries like Cyprus, Greece, and Turkey, where it is due to homozygosity to other types of a° defects, like the Mediterranean deletion (--^MED^) and the 20.5 kb α-thalassemia deletion (-(α)^20.5^).^7^–^9^ Hemoglobin Barts hydrops fetalis, however, has not been reported, to our knowledge, in the Middle Eastern Arab countries, because of the scarcity of the Mediterranean deletion or other α° defects.[10,11] This report of hemoglobin Barts hydrops fetalis from Iraq is the first report from this country. It follows a recent report on the molecular basis of α thalassemia in northern Iraq, which documented that around a quarter of the α-thalassemia determinants were due to the Mediterranean deletion (--^MED^).^12^ Thus, it would not be unexpected that the couple who are both heterozygous (αα/--^MED^), would be at risk of getting a fetus who is homozygous for this deletion (--^MED^/--^MED^).

Except for the most recent publication,[12] molecular defects responsible for α-thalassemia had not been previously studied in Iraq. Techniques for its molecular characterization only very recently became available in this country, in a diagnostic setting. Thus, it may not be unexpected to detect further cases of hemoglobin Barts hydrops fetalis in northern Iraq in the future, especially in view of the relative significant contribution of the (--^MED^) deletions to a-thalassemia determinants in this part of the country. Accordingly, it maybe prudent to consider offering carriers of the Mediterranean deletion detected by premarital screening (currently practiced in this part of the country), the choice of prenatal diagnosis of this ultimately fatal condition by chorionic villus sampling at 10-12 weeks gestation.
